# Well-being and Coping with Stress Among Russian Adolescents in Different Educational Environments

**DOI:** 10.11621/pir.2021.0305

**Published:** 2021-09-30

**Authors:** Kirill D. Khlomov, Alexandra A. Bochaver, Alexey A. Korneev

**Affiliations:** a Russian Presidential Academy of National Economy and Public Administration, Moscow, Russia; b HSE University, Moscow, Russia; c Faculty of Psychology, Lomonosov Moscow State University, Moscow, Russia; d Institute of Study of Childhood, Family and Education of the RAE, Moscow, Russia

**Keywords:** Adolescence, coping strategies, educational environment, school, well-being

## Abstract

**Background:**

The school environment influences a child’s well-being in different ways, not only by education but also by forming social roles, habits, and stress responses. It provides the sources of stress as well as the sources of resilience.

**Objective:**

This study examines the variety of coping strategies of adolescents attending different educational institutions and the different trajectories in the adaptation process in different educational environments.

**Design:**

This paper examined the coping strategies, optimism, and subjective well-being of students in different educational environments. Three schools were represented, and 646 adolescents between 12–17 years old participated in the study. The measures included the Ways of Coping Checklist, The Life Orientation Test, and The Warwick-Edinburgh Mental Well-Being Scale.

**Results:**

Coping strategies used by students attending different schools significantly differ in their intensiveness of use and age distribution. However, optimism and subjective well-being are higher among older adolescents and do not depend on the educational environment.

**Conclusion:**

The differences in the coping strategies preferred by the adolescents in different types of schools reflect their adaptation to the different environmental demands, which is confirmed by the same level of subjective well-being and optimism in different environments. However, their repertoires of coping strategies are not analogous: the students in high-rated schools use more various and more constructive coping strategies than students in low-rated schools. We may assume that their resilience and ability to cope with stress outside of school may also differ, which, in turn, can influence their further life trajectories and ability to cope with difficulties in life, perpetuating existing social inequality. Early and middle adolescents in all types of schools show a lower level of well-being and optimism than older students, which may indicate their higher psychological vulnerability and need for adult attention and support compared to older adolescents.

## Introduction

The increase in depression among youth around the world (WHO, 2019), combined with high rates of bullying and cyberbullying in Russian schools (OECD, 2019), along with an increase in cases of school shootings^1^, raise questions about Russian school students’ well-being and resilience towards stress. Schools have strong differences in their educational achievements, school climate, reputation, and history in Russia despite the reforms of 2012, whereby particular schools integrated into large “educational complexes” to create uniform educational conditions, including up to 20 buildings. This study aims to compare different educational environments from the perspectives of the preferred coping strategies and well-being of students attending these institutions and to discuss the adaptations implemented by these ado lescents.

The school environment may influence a child in different ways, not only by education directly but also by forming social roles, habits, behavioral, and communication norms (Crosnoe, 2011). The school environment influences psychological well-being (Tian, Zhao, & Huebner, 2015), health (Symonds, Dietrich, Chow, & Salmela-Aro, 2016), and social adaptation (Wolke & Lereya, 2015) in students. Secure school connectedness, positive teacher influences, supportive peers, and opportunities for academic and other success appear to relate positively to adolescent resilience (Olsson, Bond, Burns, Vella-Brodrick, & Sawyer, 2003) and work as a protective factor mitigating against risks (Oldfield, Stevenson, Ortiz, & Haley, 2018). A higher level of school engagement is related to a higher level of well-being among school students (Tian et al., 2015; Cadime et al., 2016).

At the same time, schools provide a wide range of different stress situations, from the routine difficult communicative challenges to the hard exams. For example, the *Stress in America* survey by the American Psychological Association (2014) suggested an unhealthy level of stress among adolescents, who reported that school (83%), and gaining entry into university or deciding what to do after secondary school (69%), were the two most common sources of stress at this age. Being bullied at school leads to a decrease in somatic and emotional well-being (Hellfeldt, Gill, & Johansson, 2016) and harms performance (Oliveira, de Menezes, Irffi, & Oliveira, 2018; Stavrinides, Georgiou, Nikiforou, & Kiteri, 2011).

Therefore, school is one of the heavyweight environments where stress has a place and where students form, pilot, and master their coping strategies through their social adaptation. Surprisingly, there is little known about the role of the school in developing coping strategies among students. Coping behaviour is an adaptive process that includes “cognitive and behavioral efforts to master, tolerate, or reduce external and internal demands and conflicts” (Folkman & Lazarus, 1980, p. 223) by utilizing personal and social resources to solve the stressful problem or manage the individual’s negative emotional reactions (Folkman & Lazarus, 1985; Compas, Jaser, Dunbar, Watson, Bettis, Gruhn, & Willians, 2014; Losoya, Eisen-berg, & Fabes, 1998).

There are several views on the role of coping in the overall resilience and wellbeing of a person. We use an approach, according to which, coping as a fundamental adaptive process integrates the development of stress reactivity with the emotional, motivational, behavioral, and other forms of regulation that are mobilized by stressful events. Resilience may be viewed as a dynamic adaptation process to a risk setting that involves interaction between a range of risk and protective factors, from the individual to the social (Olsson et al., 2003). It results from the interaction between a child’s stress reaction and the environmental response to this reaction (Zimmer-Gembeck & Skinner, 2016). We suppose that in different environments, different coping strategies may be supported and termed as socially acceptable be-haviour.

Although there is no universal consensus regarding the classification of coping strategies, most studies are consistent in the associations between types of coping and social adaptation. Problem solving, planning, positive reappraisal, emotional expression, support and information seeking, and problem-focused support are predominantly associated with a lower level of internalizing problems, externalizing behaviour problems, and better social competence. Coping strategies like avoidance, self-blame, venting, and rumination are generally associated with more internalizing and externalizing symptoms, and poorer adjustment and social competence (Losoya et al., 1998; Horwitz, Hill, & King, 2011; Rafnsson, Johnson, & Windle, 2006; Li, DiGiuseppe, & Froh, 2006; Zimmer-Gembeck & Skinner, 2016; Lengua & Storm-shak, 2000; Fields, & Prinz, 1997; Compas, Connor-Smith, Saltzman, Thomsen, & Wadsworth, 2001; Clarke, 2006; Krattenmacher et al., 2013).

Although many studies focus on school-related stress and coping (*e.g.*, Yuan, Zhang & Fu, 2017; Warren, 2000; Paul, Smith & Blumberg, 2012; Harper et al., 2012; Ganim, Frydenberg, 2006), they predominantly focus on coping with an event or phenomenon including exams, bullying, cyberbullying, and switching to remote learning. There is clearly insufficient data on how school characteristics themselves relate to coping strategies preferred by students.

This study aims to explore how the characteristics of the school environment, summarized in the unified city school ranking, are related to both the coping strategies used by students and their psychological well-being. We used the psychological well-being scale to measure the level of subjective well-being as a direct indicator and optimism as an indirect indicator of well-being, reflecting confidence towards the world and the predominance of positive expectations (*e.g.*, Scheier, Carver, & Bridges, 2001). Our hypotheses are as follows:

1. different preferred coping strategies are typical for students in different educational environments;
2. in different educational environments, the level of well-being of students differs.

## Methods

### Participants

The a-priori power analysis showed that if we set medium effect size (η2 = 0.0625), α = 0.05, power = 0.95, for our design, the sufficient sample size is equal to 235 or more participants. The sample consisted of 646 adolescents, aged 12–17 years, mean age — 15.28 years old. We divided the sample into three age groups (12–13 years, 108 participants, 55 males, 53 — females; 14–15 years, 269 participants, 131 males, 138 females; 16–17 years, 269 participants, 137 males, 132 females) (see *Table 1*).

**Table 1:** Number of students in age and school groups

Age (years)	1^st^ group	2^nd^ group	3^rd^ group
12–13	11	50	47
14–15	57	60	152
16–17	154	51	64
Totals (*N* = 646)	222	161	263

### Procedure

To compare the students’ characteristics in different educational environments, we organized a sample in a special way^2^. When choosing schools for research, we relied on the Moscow Department of Education ranking position. This ranking is based on a range of criteria that include the results of the unified state exam and state fi- nal attestation of students, success in subject and cross-curriculum tests, participation in academic competitions, and the event of non-performance offenses. We were interested in schools from the top 25% of the ranking, from the bottom 25%, and schools that occupy the middle 20%. Despite the Department of Education’s attempts to reduce educational inequality and make all schools uniform, each school usually has its own unique history and reputation. They differ in the level of selectivity, socioeconomic characteristics of school students’ families, the qualifications of teachers, and the strategies of intra-school psychological services.

The three basic strategies of school psychological services function may be described as follows: 1) “Disaster recovery”: psychological rescue actions usually follow incidents such as fights, substance use, and crimes, in collaboration with the police, medical personnel, the commission for juvenile affairs and protection of their rights. This is commonly associated with the schools from the bottom 25%; 2) “Caring for the future”: different prevention programs are conducted and demanded from external specialists, but the current psychological problems are not always addressed. This strategy is used in schools that occupy the middle 20%; and 3) “System approach”: multidirectional psychological work includes training, counselling, prevention programs, and education and support for the teachers and the parents. It is implemented by the schools from the top 25% of the ranking.

These indicators are mostly directly or implicitly reflected in the school’s ranking. Children who study in schools with different ratings are in distinctly different social conditions, with different norms and requirements.

Nine educational institutions were chosen as platforms for the research, and they were combined into three groups of three institutions.

#### 1^st^ group

A school with a low ranking and two institutions of secondary special education (colleges), characterized by low educational achievement and unsafe be-haviour among the students (*N* = 222 students).

#### 2^nd^ group

Three schools with an average ranking (*N* = 161 students).

#### 3^rd^ group

Three schools with a high ranking (*N* = 263 students).

### Questionnaires

The participants completed three questionnaires in the Russian adaptation:

1. Ways of Coping Checklist by Folkman and Lazarus which includes scales of Confrontation (Chronbach’s α = 0.51, McDonald’s ω = 0.51 in our sample), Distancing (α = 0.52, ω = 0.54), Self-Control (α = 0.45, ω = 0.46), Social Support Seeking (α = 0.59, ω = 0.61), Accepting Responsibility (α = 0.51, ω = 0.53), Escape-Avoidance (α = 0.56, ω = 0.56), Planning of Problem Solving (α = 0.69, ω = 0.70), and Positive Reappraisal (α = 0.62, ω = 0.64) (Kryukova & Kuftyak, 2007). The responses to items were presented on a Likert scale from 0 to 3;
2. The Life Orientation Test by Carver & Scheier (Gordeeva, Sychev, & Osin, 2010). We use the scale of optimism in our study (Chronbach’s α = 0.78, McDonald’s ω = 0.79 in our sample). The responses to items were presented on a Likert scale from 0 to 4;
3. The Warwick-Edinburgh Mental Well-Being Scale (Tennant et al., 2007, Chronbach’s α = 0.88, McDonald’s ω = 0.88 in our sample). The responses to items were presented on a Likert scale from 1 to 5.
  For the analysis of coping strategies, a two-way MANOVA analysis was conducted (we used age group and school group as factors, and all scales of the Ways of Coping Checklist as outcomes). To analyze the separated effects of age and school groups on various scales, a series of ANOVA’s were used.

## Results

In *Table 2*, the general results of the MANOVA are presented. There is only one significant effect of school groups. The coping strategies, in general, are more pronounced in the 3rd group.

**Table 2:** MANOVA results

Factor	Df	F	η^2^	P
	*General*			
Age	16, 1262	1.382	0.016	0.142
Type of school	16, 1262	2.122	0.026	0.006
Age X Type of school	32, 2532	1.186	0.015	0.219

**Figure 1.**
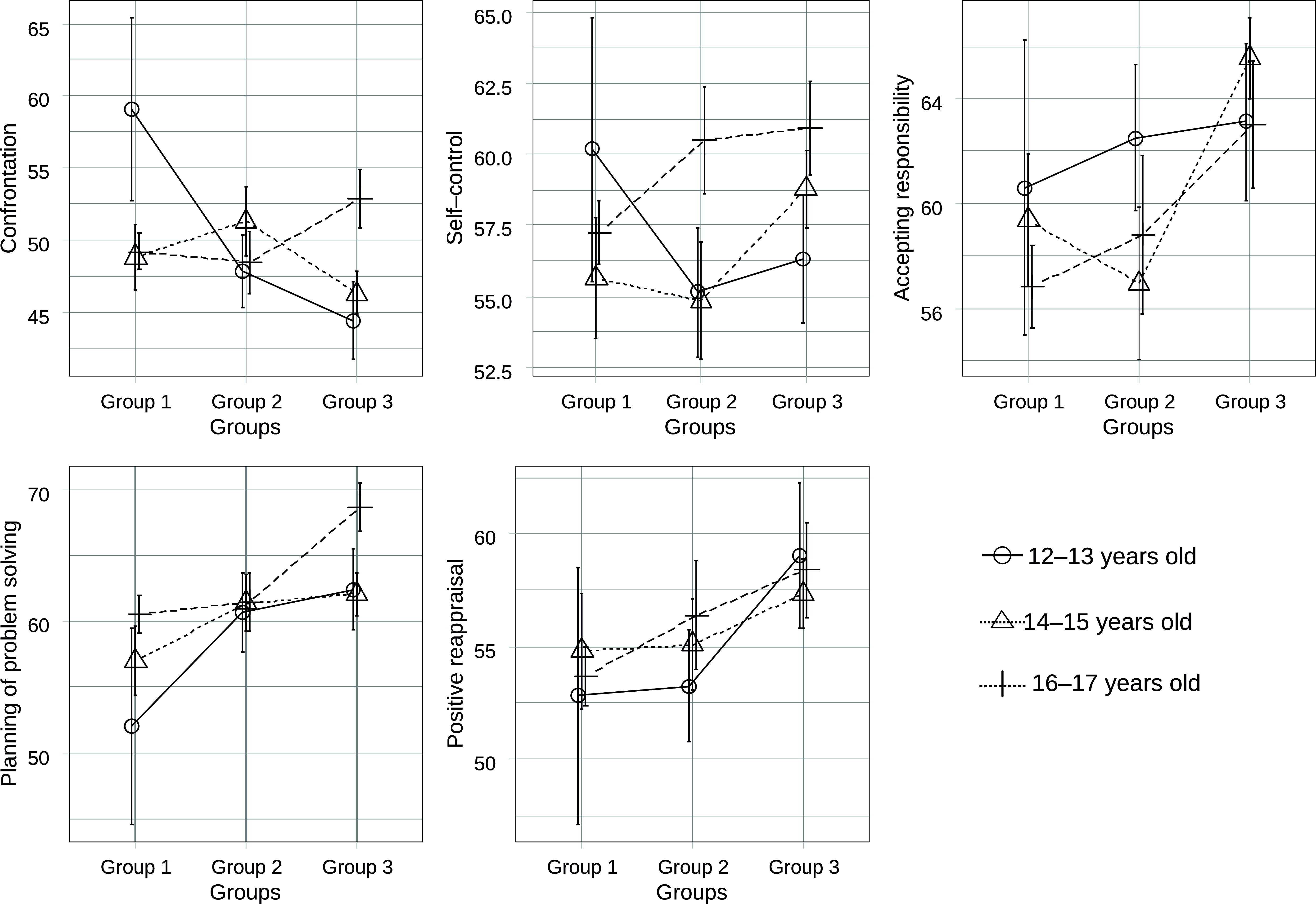
Coping strategies of the students in the three groups of institutions (only significant results are presented).

Besides this general effect, more interesting and meaningful are the effects of age and school groups on separate coping strategies. Table 3 presents significant differ-ences in adolescents’ coping strategies with stress situations from different educational environments (see also *Figure 1*). As we conducted a series of ANOVA’s, we included raw p-value and adjusted p-values (we used the FDR method, Benjamini, Hochberg, 1995) in the table. Below we discuss unadjusted p-values since we want to estimate general tendencies that can be investigated in detail in further studies.

**Table 3:** ANOVA results for coping strategies scales with significant effect

Factor	Df	F	η^2^	P	P adjusted
*Confrontation*					
Age	2, 637	1.198	0.002	0.302	0.605
Type of school	2, 637	0.557	0.002	0.573	0.573
*Age X Type of school*	**4, 637**	**2.923**	**0.018**	**0.021**	*0.164*
*Self-Control*					
Age	2, 637	1.302	0.007	0.273	0.605
Type of school	2, 637	2.41	0.008	0.091	*0.181*
Age X Type of school	4, 637	0.852	0.005	0.493	0.875
*Accepting Responsibility*					
Age	2, 637	2.745	0.002	0.065	*0.520*
Type of school	**2, 637**	**5.6937**	**0.019**	**0.003**	**0.014**
Age X Type of school	4, 637	0.69563	0.004	0.595	0.875
*Planning of Problem Solving*					
Age	2, 637	0.795	0.009	0.452	0.723
Type of school	**2, 637**	**5.804**	**0.018**	**0.003**	**0.014**
Age X Type of school	4, 637	0.322	0.002	0.863	0.881
*Positive Reappraisal*					
Age	2, 637	0.208	<0.001	0.812	0.812
Type of school	**2, 637**	**3.129**	**0.010**	**0.044**	0.118
Age X Type of school	4, 637	0.713	0.004	0.583	0.875

In the institutions of the 3^rd^ group, the strategies of Planning of Problem Solving, Positive Reappraisal, Taking Responsibility, and Self-Control (sub significantly) have a stronger representation compared to the other groups. These strategies are very important for both coping functions: stress situation transformation and emotional regulation.

In the institutions of the 1^st^ group, Confrontation (among younger teenagers) has a higher representation.

The 2^nd^ group shows a medium level of coping strategies.

There are no differences between groups on the scales of Distancing, Escape-Avoidance, and Social Support Seeking strategies. Escape-Avoidance coping is predominantly discussed as disengagement coping and typically related to the higher number of internalizing problems (Compas et al., 2001). Distancing and Social Support Seeking is usually classified as emotional-focused coping, which is aimed at emotional expression and doesn’t change the problem situation. However, social support may have different forms and functions (Compas et al., 2001).

**Figure 2.**
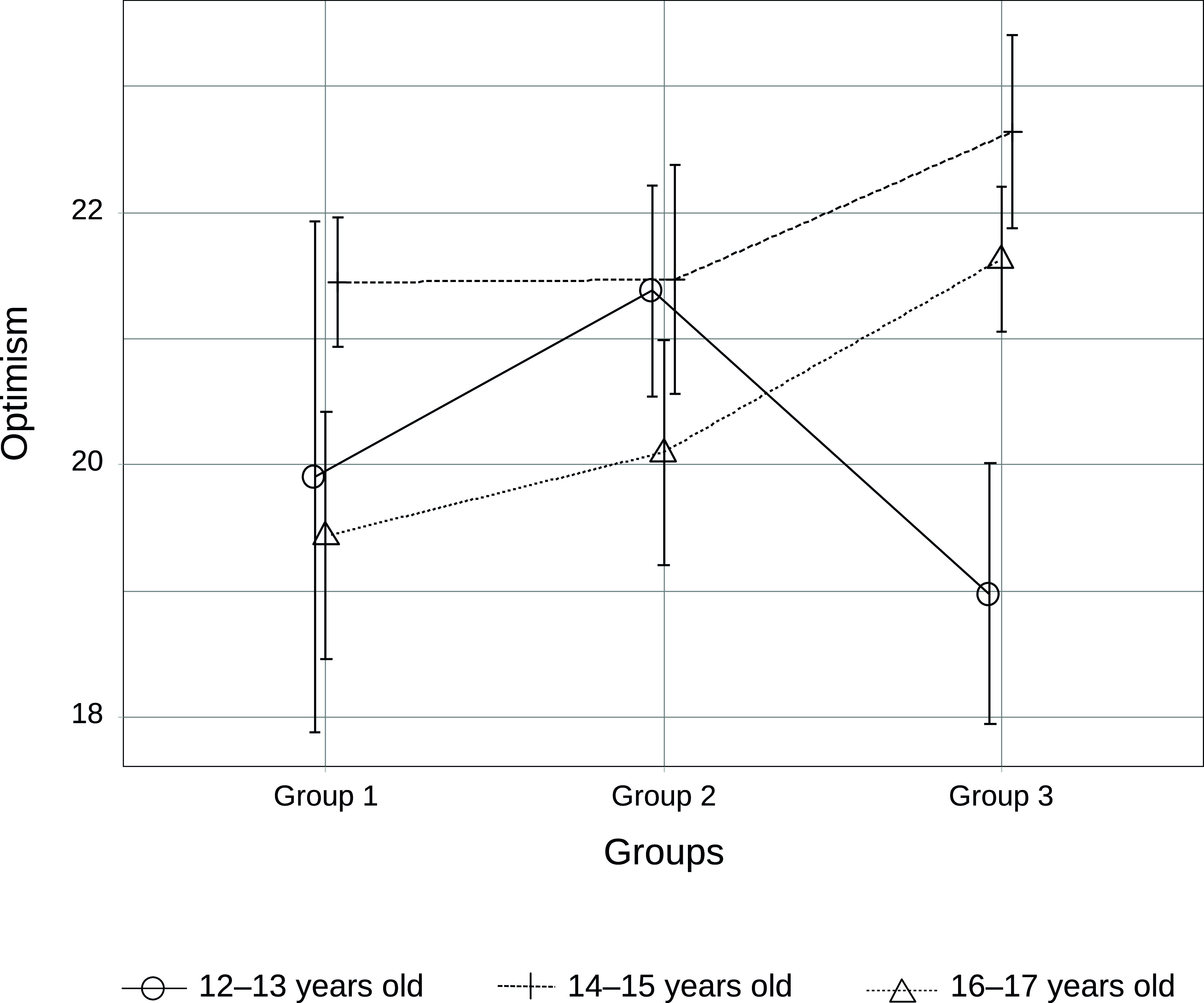
Level of optimism of the students in three groups of institutions.

**Figure 3.**
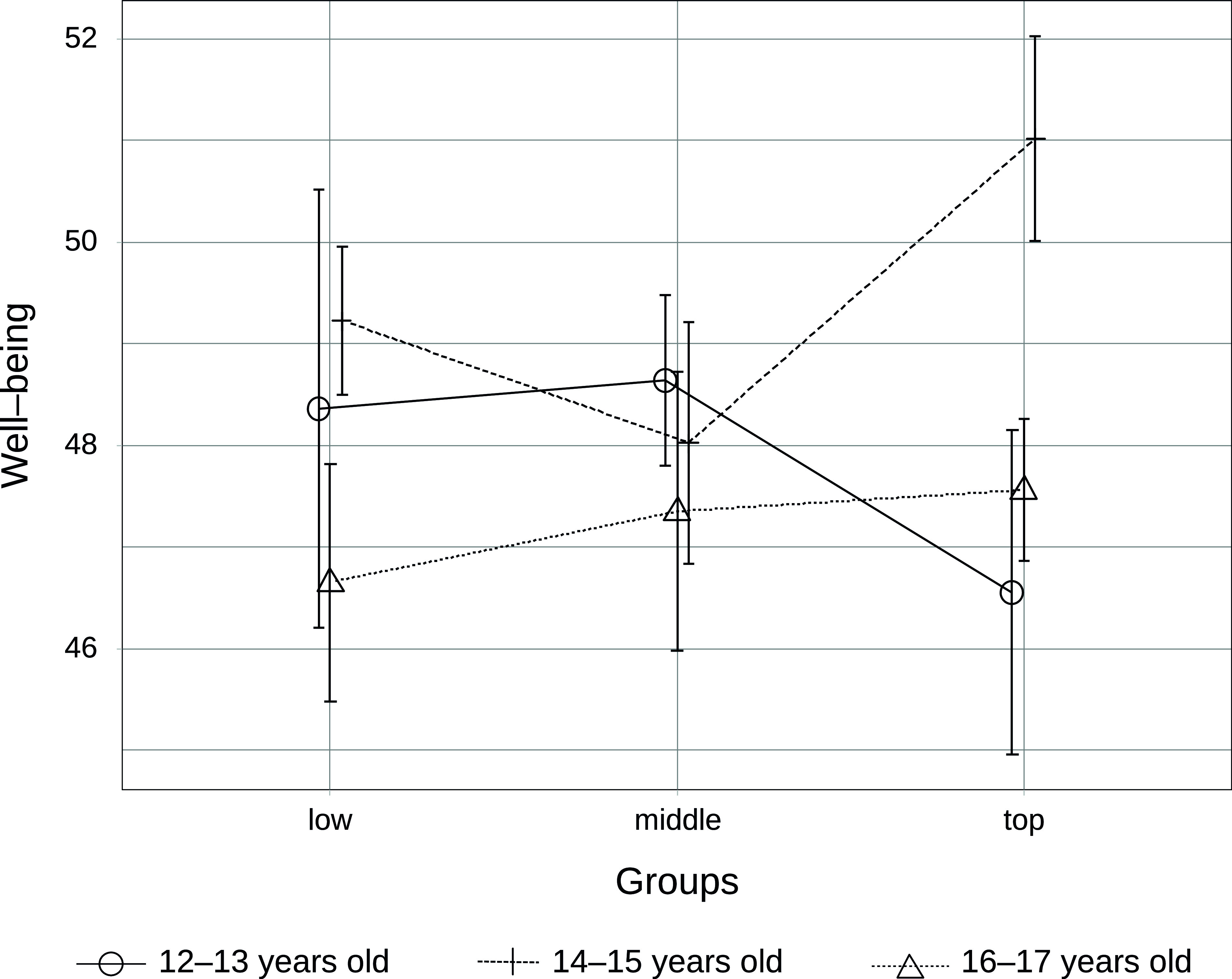
Level of mental well-being of the students in three groups of institutions.

The results of the ANOVA for scales of Optimism and Well-being are presented in *Table 4.* Results, depicted in *Figure 2* and *Figure 3*, demonstrate a significant increase in optimism (see *Figure 2)* and mental well-being (see *Figure 3*) among respondents over time, with no difference between school types.

**Table 4:** ANOVA results for optimism and well-being

Factor	Df	F	η^2^	P
*Optimism*					
Age	**2, 637**	**4.36**	**0.017**	**0.013**
Type of school	2, 637	1.25	0.003	0.286
Age X Type of school	4, 637	1.78	0.011	0.131
*Mental Well-Being*					
Age	**2, 637**	**4.04**	**0.013**	**0.018**
Type of school	2, 637	0.44	0.001	0.708
Age X Type of school	4, 637	1.15	0.007	0.333

## Discussion

The data show several important results. Our first hypothesis is confirmed: there are significantly different coping strategies preferred by students in different types of schools and, contribute to their well-being as optimal for the environmental demands. In the 1st group, Confrontational behaviour is higher among the younger and lower among the older adolescents; such coping strategies as Problem Solving, Positive Reappraisal, Self-Control, and Accessing Responsibility are significantly more frequently presented in the 3rd group. These differences confirm that students’ adaptation to different environments is diverging. In the 3rd group, the coping strategy repertoire is broader, indicating that these students are more competent in problem-solving (which is steadily associated with lower externalizing and internalizing problems) (Compas et al., 2001), self-regulation (Self-Control scale), rethinking their experiences (Positive Reappraisal scale), and responsible perception of the situation (Accepting Responsibility scale), than in the institutions of the 1st and 2nd groups. Confrontation coping is more widely used by younger adolescents in the institutions of the 1st group, but the older students of the 3rd group use it more intensively than in the 1st group. The schools of the 2nd group occupy the middle position between the 1st and 3rd groups.

Since coping behaviour is sensitive to environmental responses, we can assume that different behavioral patterns are supported in schools of different groups through observational social learning, adult encouragement, and norms of socially acceptable behaviour realized in the school. Seemingly, in the 1st group, the predominantly obedient, manageable behaviour with the external locus of control among the students is supported. Partly, it may be related to the characteristics of the students contingent (children with low academic achievement and motivation, and externalizing behav-iour problems may provoke stricter responses by the teachers). Still, it is remarkable that neither aggression nor awareness are supported. The behavioural repertoire increases slightly, but students’ manageability and controllability seem to develop and get support.

In the 3rd group, we can assume the positive environmental response towards variable behaviour, particularly with a high level of self-control and problem-solving planning. Environmental tolerance to adolescents’ confrontational behaviour (the Confrontation scores is higher in older students), in combination with the support of responsibility, the ability to plan their actions and predict the consequences, along with self-control, may promote personal autonomy development.

The schools of the 2nd group have a position between the 1st and 3rd groups; in figures 1, 2, and 3, their profile looks closer to the profile of the 1st group, but there is no statistical confirmation now, and this similarity requires further studies.

These results partly correspond with the previous studies of coping within the school environment. For example, academic stress-coping strategies may be predicted by students’ thinking styles (Yuan, Zhang & Fu, 2017). These may be developed differently in different environments; positive attitudes to school are predicted by a low level of school-related stress, a high level of well-being, and different constellations of the coping strategies for males and females (Ganim, Frydenberg, 2006). Harper et al. (2012) showed that coping effectiveness suppresses the effects of peer victimization on perceived school safety. However, there is a lack of research on the coping behaviour of schoolchildren in the context of different educational environments. This explains the novelty of this work, but at the same time, it makes the work less complete and requires further research.

Surprisingly, the second hypothesis isn’t confirmed. There are no significant differences in well-being and optimism in educational environments. However, there are differences associated with age: between the ages of 12–15 years old, the adolescents demonstrate a significantly lower level of well-being than older students, which means that this age group is especially vulnerable to the different stressors. An increase of the mental well-being and optimism from younger to older adolescence is shown in other studies (*e.g.,* Salmela-Aro & Tuominen-Soini, 2010; Sanders et al., 2015), and this tendency may reflect maturation processes and an increase of adjustment to the present conditions and successful coping with stress that manifests, for instance, in improving well-being.

The findings show the splitting of the educational strategies and results. In the educational environments where the lower educational level is dominant, and harder psychological problems are noticed (the 1st group), obedience and manageability among students are fostered. In environments with higher academic achievements and attention paid to the psychological problems (the 3rd group), more complex and versatile behaviour is supported.

## Conclusion

Our results show that adolescents adapt to their environment over time and build up their resilience in various conditions. Early and middle adolescence seems to be the period of higher vulnerability among adolescents and requires the most attention and support provided by the social environment.

Despite a key feature of adolescence being a growing autonomy, our findings show that, in only certain schools, personal autonomy was encouraged and fostered. Alternatively, in other schools, obedience, but not personal autonomy, is encouraged. These diffferences in schools indirectly support the different patterns of adaptation towards complicated social conditions. They foster and increase social inequality and a split in the prospective personal maturity in students graduating from the different school groups.

According to the demands-resource model, the environment provides resources and simultaneously imposes demands on students, including effort and usually have physical and psychological costs. The resources can help to diminish the stress induced by this effort and aid the individuals in fulfilling their personal needs and boost their positive adjustment. A mismatch between the students’ developmental needs and the school environment can provoke different psychological and mental health problems (Symonds et al., 2016; Cadime et al., 2016). Our study shows some directions in the differences in the efforts made by students to cope with stress, but many questions require further research. In particular, in the future, it is important to make a deeper assessment of the school climate, norms and values within each organization, as well as to study the coping strategies used by students in a long-term study.

## Limitations

In this study, there is an uncontrollable factor of individual differences between age groups. The formal rating criterion chosen for the sample formation does not give a complete picture of the features of the educational environment. Future research should include a longitudinal study to avoid these limitations and pay more attention to the students’ individual differences and school environment assessment.

## Footnotes

1 *e.g.* Russia school shooting: Children and teacher killed in Kazan https://www.bbc.com/news/world-europe-57069589

2 This article is also devoted to the results obtained from the same sample: Khlomov, K.D., Bocha-ver, A.A., Korneev A.A. (2020). Coping Strategies of Adolescents and Educational Environment. *Social psychology and society, 11*(2), 180–199. https://doi.org/10.17759/sps.2020110211
